# Supplementary material for: Impaired telomere integrity and rRNA biogenesis in PARN‐deficient patients and knock‐out models
Source: EMBO Mol Med. 2019 Jun 6;11(7):e10201. doi: 10.15252/emmm.201810201 (PMC6609912; doi:10.15252/emmm.201810201)
Supplement: Supplementary file 1 — Appendix [file EMMM-11-e10201-s001.pdf]

Appendix files

Impaired telomere integrity and rRNA biogenesis in PARN-deficient patients and knock-out models

Table of content:

Appendix Figure S1…………………………………………..page 2
Appendix Figure S2…………………………………………..page 3
Appendix Figure S3…………………………………………..page 4
Appendix Figure S4…………………………………………..page 5
Appendix Figure S5…………………………………………..page 6
Appendix Figure S6…………………………………………..page 7
Appendix Table S1…………………………………………....page 8
Appendix Table S2……………………………………………page 9
Supplementary References……………………………………page 10

Appendix Figure S1

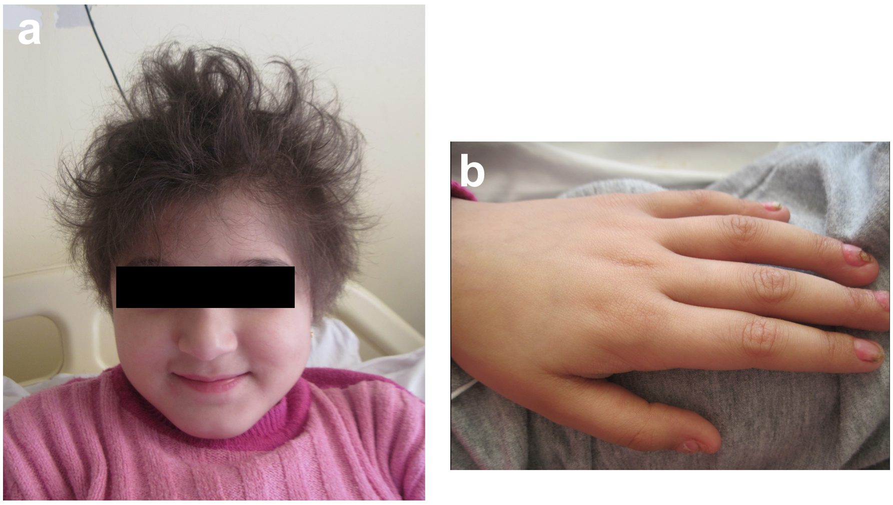

Appendix Figure S1. Pictures showing coarse hairs **(a)** and nail dystrophy **(b)** in individual P1.

Appendix Figure S2

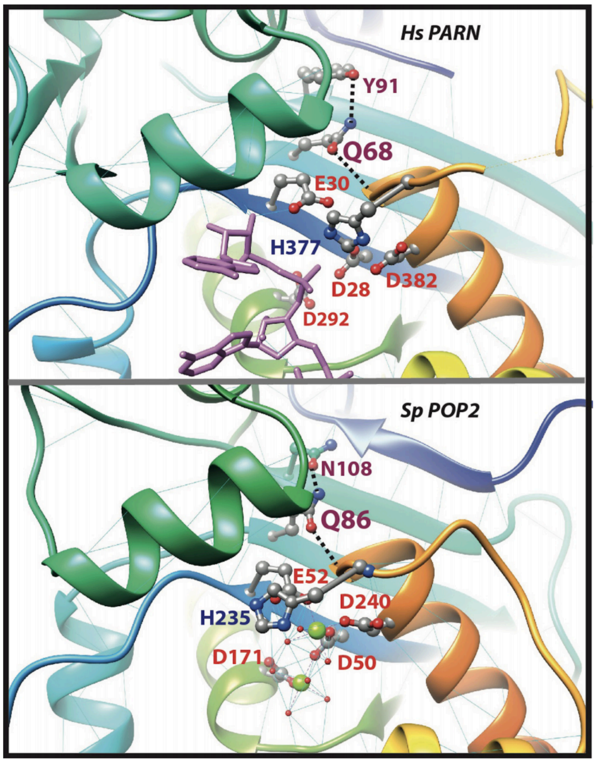

Appendix Figure S2. The active site of human PARN in complex with RNA (purple) (upper panel, pdb 2A1R (Wu et al, 2005)) is compared to the active site of *S. pombe* POP2 (lower panel, pdb 2P51 (Jonstrup et al, 2007)). Mg^{++}: green balls; water: red balls. Three aspartate (Hs PARN D28, D292, D382; Sp POP2 D50, D171, D240) and one glutamate (Hs PARN E30; Sp POP2 E52) residues play crucial roles for the hydrolytic reaction mechanism of the DEDDh subfamily of nucleases as the four acidic residues, by coordinating two divalent metal ions, ensure both the substrate binding and the interaction with a molecule of water essential for hydrolysis. The Nε2 atom of the conserved glutamine (Hs PARN Q68; Sp POP2 Q86) makes an H-bond with the oxygen atom of an amino acid of the neighbour β-strand (Hs PARN Y91 OH; Sp POP2 N108 Oδ1), whereas the Oε1 atom of the same glutamine residue is H-bonded to the N atom of the α-helix N-cap (Hs PARN A379; Sp POP2 A237), located between the catalytic histidine (Hs PARN H377; Sp POP2 H235) and the last aspartic acid (Hs PARN D382; Sp POP2 D240). This last H-bond is likely to play a crucial role in the configuration of the active site, and the p.Gln68His mutation is thus predicted to disturb catalytic activity. Figure made using Chimera (Pettersen et al, 2004).

Appendix Figure S3

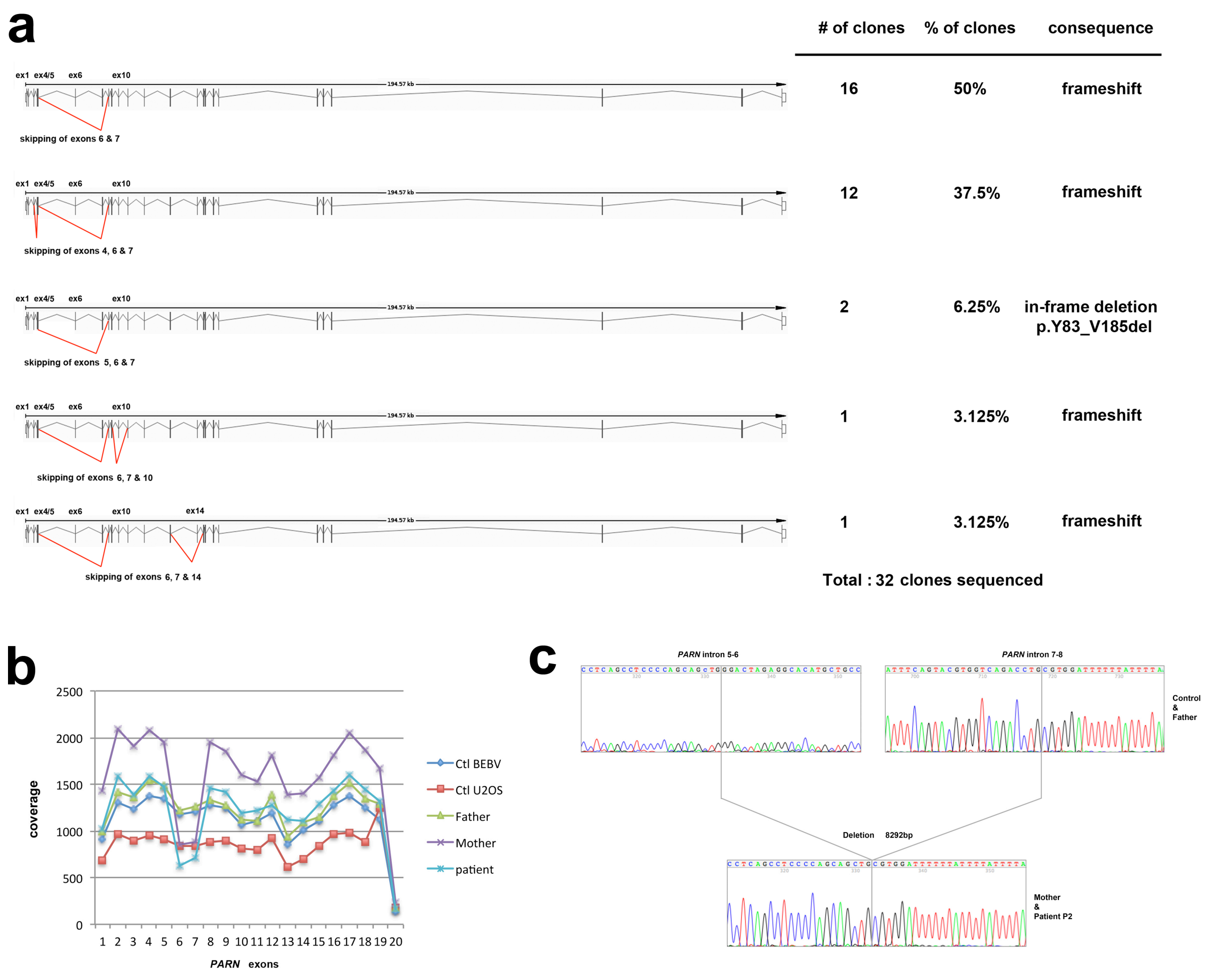

Appendix Figure S3. (a) Splicing aberrations in *PARN* transcripts identified in P2's cells. *PARN* cDNA in patient's cells was PCR amplified, cloned and sequenced. The main splicing events and their frequencies are indicated. 32 independent clones were analyzed. **(b)** Coverage analysis performed by high throughput sequencing of the whole *PARN* gene sequence (promoter, introns, exons) revealed a two-fold reduction of a genomic region encompassing the *PARN* exons 6 and 7 in P2 and her mother. **(c)** Sanger sequencing of the boundaries of the deleted region in P2.

Appendix Figure S4

a

RNA guide targeting PARN exon2 : CCGACTTCTTCGCCATCGAT

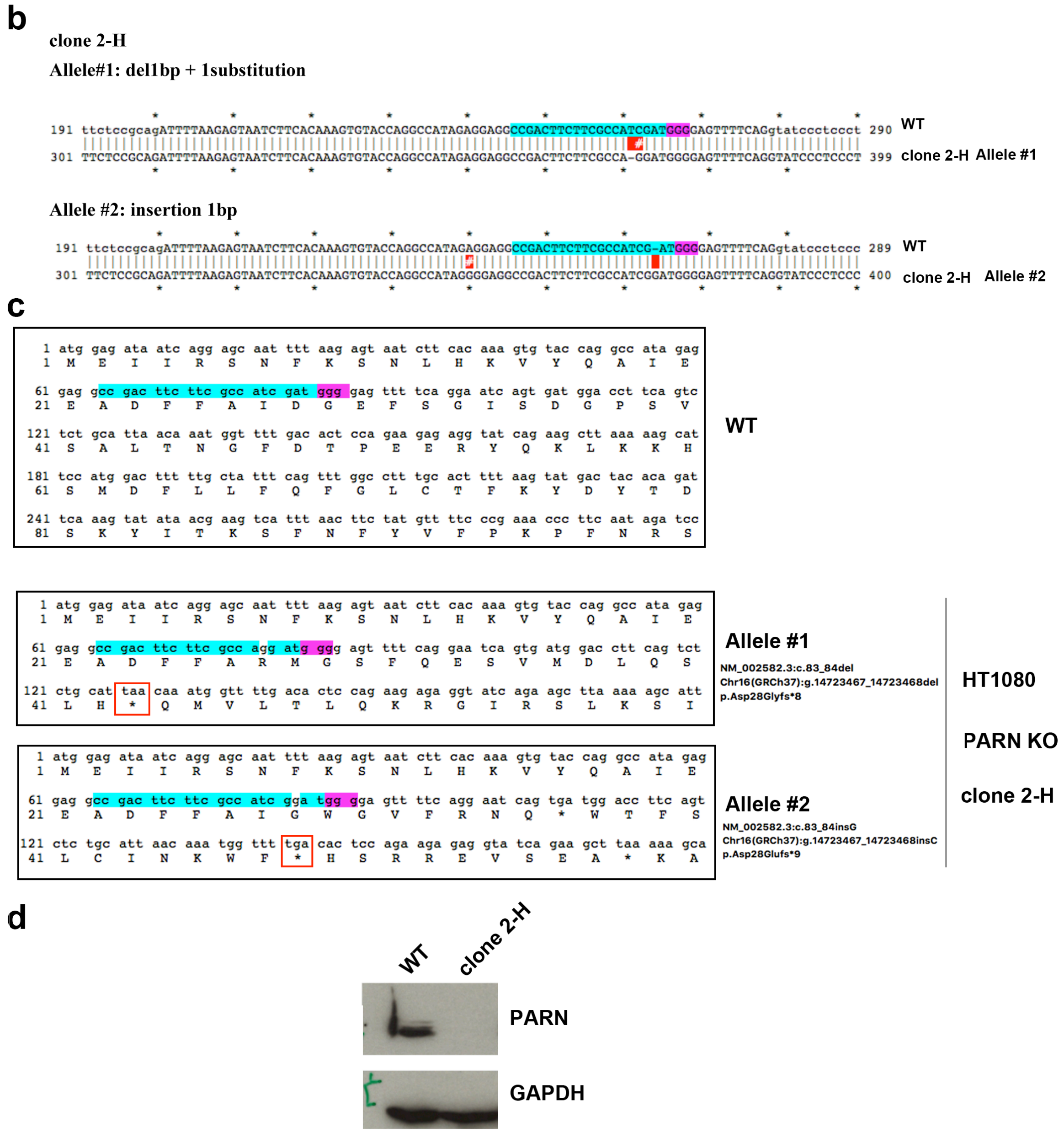

Appendix Figure S4. (a) Design of the gRNA used to create CRISPR/Cas9 mutations in the *PARN* coding sequence. **(b)** The HT1080^{PARNKO} clone 2-H harbors a 1-bp deletion and a substitution resulting in a frameshift on one allele and 1bp –insertion, also resulting in a frameshift, on the other allele. The blue sequence represents the locus targeted by the gRNA. The pink sequence represents the PAM. **(c)** Premature stop codon generated by both mutations are indicated by red boxes. **(d)** Western Blot analysis confirming the PARN loss of function in HT1080^{PARNKO} clone 2-H.

Appendix Figure S5

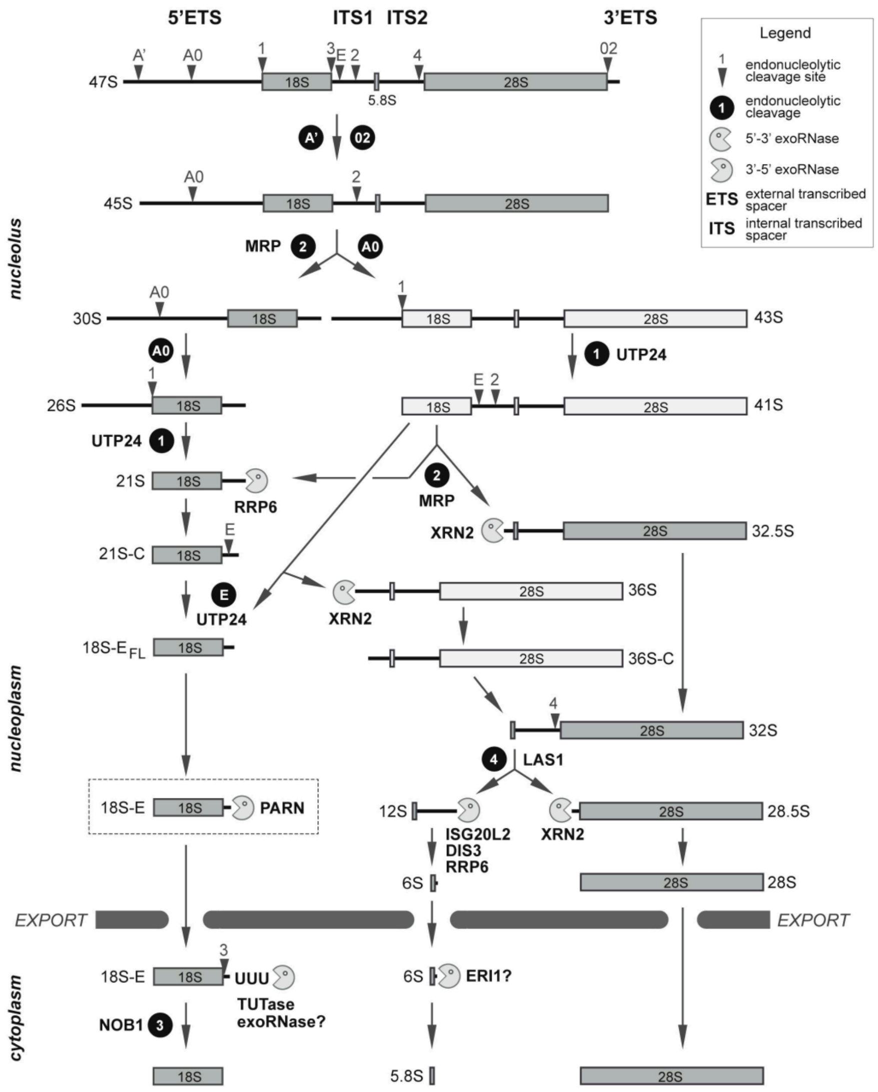

Appendix Figure S5. Ribosomal RNA processing in human cells. Three of the four rRNAs forming the backbones of the small and large ribosomal subunits arise from a long primary transcript (47S pre-rRNA), in which 18S, 5.8S and 28S rRNA sequences are flanked by external (5'ETS, 3'ETS) and internal transcribed spacers (ITS1, ITS2). These rRNAs are progressively released owing to endo- and exoribonucleases. Arrows displayed along the rRNA precursor sequences point out the positions of endonucleolytic cleavage sites. The sequential order of these cleavages defines major and minor, alternative pathways, giving rise to abundant precursors (depicted in grey) or rarer pre-rRNA species (depicted in white). When identified, endoribonucleases are labelled in black, and 5'-3' or 3'-5' exoribonucleases in grey. Question marks refer to putative enzymatic activities deduced from 3'RACE data (Montellese et al, 2017; O'Donohue et al, 2010), or to an RNAse described in mouse whose human ortholog could fulfill similar functions (Ansel et al, 2008).

Appendix Figure S6

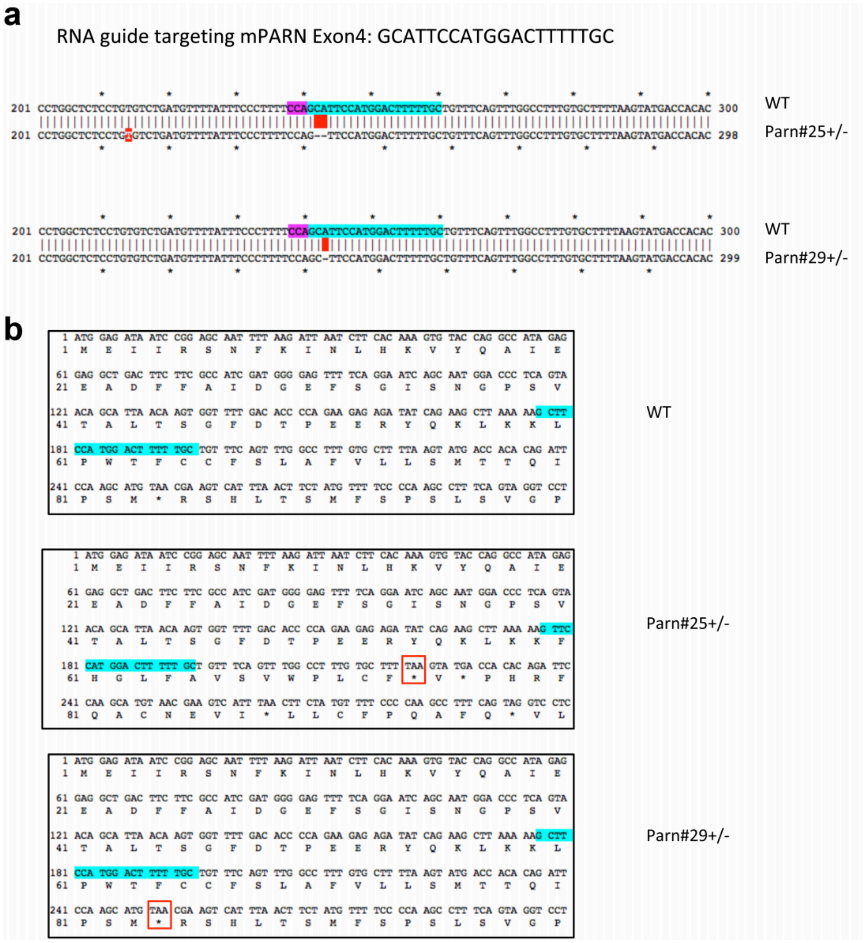

Appendix Figure S6. (a) Design of the gRNA used to create CRISPR/Cas9 mutations in the mouse *Parn* gene. The blue sequence represents the locus targeted by the gRNA. The pink sequence represents the PAM. **(b)** The Parn#25+/- and Parn#29+/- animal cell lines harbor, respectively, a 2-bp and a 1-bp deletion on one allele, resulting in frameshifts and premature stop codons indicated by red boxes.

Appendix Table S1. List of predicted off-target loci sequenced and found in Parn#25+/- and Parn#29+/- animals.

***PAM* + gRNA sequence**
cca gcattccatggactttttgc

gene name	position	number of mismatches	off-target sequence	Sanger sequence
Gm17441/Tcte1	chr17:45,679,275-45,679,297	3	*cc**t*** gca**c**tcca**g**gact**c**tttgc	WT
Abca1	chr4:53,043,874-53,043,896	4	*cc**c*** **t**cattcca**a**g**c**actt**t**a**t**gc	WT
Efcab5	chr11:76,916,908-76,916,930	4	*cc**t*** **cg**attccatggacttt**g**tg**t**	WT
Olfr506	chr7:115,755,996-115,756,018	4	*cca* **t**cat**c**ccatg**t**actttttt**t**c	WT
Olfr487	chr7:115,355,846-115,355,868	4	*cca* **t**cat**c**ccatg**t**actttttt**t**c	WT
Olfr478	chr7:115,175,660-115,175,682	4	*cca* **t**cat**c**ccatg**t**acttttt**c**c	WT
Garnl3	chr2:32,878,191-32,878,213	4	*cca* g**g**a**a**t**t**c**ag**ggactttttgc	WT

Appendix Table S2. Primers used in this study.

Primer name	Sequence	Reference
**ACTB-F**	TGTACGCCAACACAGTGCTG	Boros et al., 2014
**ACTB-R**	GCTGGAAGGTGGACAGCGA	Tilman et al, 2009
**hTR-F**	TTTGTCTAACCCTAACTAACTGAGAAG	Tilman et al, 2009
**hTR-R**	TTGCTCTAGAATGAACGGTGGA	Tilman et al, 2009
**POT1-F**	TCAGTCTGTTAAACTTCATTGCCC	Zaffaroni et al, 2005
**POT1-R**	TGCACCATCCTGAAAAATTATATCC	Zaffaroni et al, 2005
**TPP1-F**	CCCGCAGAGTTCTATCTCCA	Poncet et al, 2008
**TPP1-R**	GGACAGTGATAGGCCTGCAT	Poncet et al, 2008
**TRF2-F**	GACCTTCCAGCAGAAGATGC	Escoffier et al, 2005
**TRF2-R**	GTTGGAGGATTCCGTAGCTG	Escoffier et al, 2005
**TRF1-F**	CCACATGATGGAGAAAATTAAGAGTTAT	Lin et al, 2006
**TRF1-R**	TGCCGCTGCCTTCATTAGA	Lin et al, 2006
**RAP1-F**	CGGGGAACCACAGAATAAGA	Poncet et al, 2008
**RAP1-R**	CTCAGGTGTGGGTGGATCAT	Poncet et al, 2008
**TIN2-F**	GTCAGAGGCTCCTGTGGATT	Abreu et al, 2010
**TIN2-R**	CAGTGCTTTCTCCAGCTGAC	Abreu et al, 2010
**RTEL1-F**	TCCCAAAGATTATTTACGCC	Tummala et al, 2015
**RTEL1-R**	TCTGTAGATGGTTACTCTCTTG	Tummala et al, 2015
**DKC1-F**	CTGGAGTCATGAGTGAAAAG	Tummala et al, 2015
**DKC1-R**	CTCATCCTTGTGGTTATCATAC	Tummala et al, 2015
**p53-F**	GAGGTTGGCTCTGACTGTACC	Liu et al, 2015
**p53-R**	TCCGTCCCAGTAGATTACCAC	Liu et al, 2015
**p21-F**	GCAGACCAGCATGACAGATTT	Liu et al, 2013
**p21-R**	GGATTAGGGCTTCCTCTTGGA	Liu et al, 2013
**Xp-Yp-F**	GCAAAGAGTGAAAGAACGAAGCTT	Porro et al, 2010
**Xp-Yp-R**	CCCTCTGAAAGTGGACCAATCA	Porro et al, 2010
**16p-F**	TGTGTTTCAACGCTGCAACTG	Diman et al, 2016
**16p-R**	AGTTAGAACGGTTCAGTGTG	Diman et al, 2016
**5p-F**	GAGTGCATTAGCATACAGGTG	Diman et al, 2016
**5p-R**	TCCTAATGCACACGTAACAC	Diman et al, 2016
**15q-F**	CAGCGAGATTCTCCCAAGCTAAG	Porro et al, 2010
**15q-R**	AACCCTAACCACATGAGCAACG	Porro et al, 2010

Supplementary References

-Abreu E, Aritonovska E, Reichenbach P, Cristofari G, Culp B, Terns RM, Lingner J, Terns MP (2010) TIN2-tethered TPP1 recruits human telomerase to telomeres in vivo. *Mol Cell Biol* 30:2971-82

-Ansel KM, Pastor WA, Rath N, Lapan AD, Glasmacher E, Wolf C, Smith LC, Papadopoulou N, Lamperti ED, Tahiliani M, Ellwart JW, Shi Y, Kremmer E, Rao A, Heissmeyer V (2008) *Nat Struct Mol Biol* 15:523-30

-Boros J, Arnoult N, Stroobant V, Collet JF, Decottignies A (2014) Polycomb repressive complex 2 and H3K27me3 cooperate with H3K9 methylation to maintain heterochromatin protein 1α at chromatin. *Mol Cell Biol* 34:3662-74

-Diman A, Boros J, Poulain F, Rodriguez J, Purnelle M, Episkopou H, Bertrand L, Francaux M, Deldicque L, Decottignies A (2016) Nuclear respiratory factor 1 and endurance exercise promote human telomere transcription. *Sci Adv* 2:e1600031

-Escoffier E, Rezza A, Roborel de Climens A, Belleville A, Gazzolo L, Gilson E, Duc Dodon M (2005) A balanced transcription between telomerase and the telomeric DNA-binding proteins TRF1, TRF2 and Pot1 in resting, activated, HTLV-1-transformed and Tax-expressing human T lymphocytes. *Retrovirol* 2: 77

-Jonstrup AT, Andersen KR, Van LB, Brodersen DE (2007) The 1.4-Å crystal structure of the S. pombe Pop2p deadenylase subunit unveils the configuration of an active enzyme. *Nucleic Acids Res* 35: 3153-64

-Lin X, Gu J, Lu C, Spitz MR, Wu X (2006) Expression of telomere-associated genes as prognostic markers for overall survival in patients with non-small cell lung cancer. *Clin Cancer Res* 12: 572

-Liu J-L, Zeng G-Z, Liu X-L, Liu Y-Q, Hu Z-G, Liu Y, Tan N-H, Zhou G-B (2013) Small compound bigelovin exerts inhibitory effects and triggers proteolysis of E2F1 in multiple myeloma cells. *Cancer Sci* 104: 1697-704

-Liu Y, Zhang X, Han C, Wan G, Huang X, Ivan C, Jiang D, Rodriguez-Aguayo C, Lopez-Berestein G, Rao PH, Maru DM, Pahl A, He X, Sood AK, Ellis LM, Anderl J, Lu X (2015) TP53 loss creates therapeutic vulnerability in colorectal cancer. *Nature* 520:697-701

-Montellese C, Montel-Lehry N, Henras AK, Kutay U, Gleizes PE, O'Donohue MF (2017) Poly(A)-specific ribonuclease is a nuclear ribosome biogenesis factor involved in human 18S rRNA maturation. *Nucleic Acids Res* 45: 6822-36

-O'Donohue MF, Choesmel V, Faubladier M, Fichant G, Gleizes PE (2010) Functional dichotomy of ribosomal proteins during the synthesis of mammalian 40S ribosomal subunits. *J Cell Biol* 190:853-66

-Pettersen EF, Goddard TD, Huang CC, Cough GS, Greenblatt DM, Meng EC, Ferrin TE (2004) UCSF Chimera—a visualization system for exploratory research and analysis. *J Comput Chem* 25:1605-12

-Poncet D, Belleville A, t'kint de Roodenbeke C, Roborel de Climens A, Ben Simon E, Merle-Beral H, Callet-Bauchu E, Salles G, Sabatier L, Delic J, Gilson E (2008) Changes in the expression of telomere maintenance genes suggest global telomere dysfunction in B-chronic lymphocytic leukemia. *Blood* 111: 23888-91

-Porro A, Feuerhahn S, Reichenbach P, Lingner J (2010) Molecular dissection of telomeric repeat-containing RNA biogenesis unveils the presence of distinct and multiple regulatory pathways. *Mol Cell Biol* 30: 4808-17

-Tilman G, Loriot A, Van Beneden A, Arnoult N, Londono-Vallejo JA, De Smet C, Decottignies A (2009) Subtelomeric DNA hypomethylation is not required for telomeric sister chromatid exchanges in ALT cells. *Oncogene* 28: 1682-93

-Tummala H, Walne A, Collopy L, Cardoso S, de la Fuente J, Lawson S, Powell J, Cooper N, Foster A, Mohammed S, Plagnol V, Vulliamy T, Dokal I (2015) Poly(A)-specific ribonuclease deficiency impacts telomere biology and causes dyskeratosis congenita. *J Clin Invest* 125: 2151-60
-Wu M, Reuter M, Lilie H, Liu Y, Wahle E, Song H (2005) Structural insight into poly(A) binding and catalytic mechanism of human PARN. *EMBO J* 24: 4082-93
-Zaffaroni N, Villa R, Pastorino U, Cirincione R, Incarbone M, Alloisio M, Curto M, Pilotti S, Daidone MG (2005) Lack of telomerase activity in lung carcinoids is dependent on human telomerase reverse transcriptase transcription and alternative splicing is associated with long telomeres. *Clin Cancer Res* 11: 2832-9
