# Supplementary figures and images for: Impaired telomere integrity and rRNA biogenesis in PARN‐deficient patients and knock‐out models
Source: EMBO Mol Med. 2019 Jun 6;11(7):e10201. doi: 10.15252/emmm.201810201 (PMC6609912; doi:10.15252/emmm.201810201)

Figure 1G

kDa RPE1 Ctl SV P1 SV P2 SV

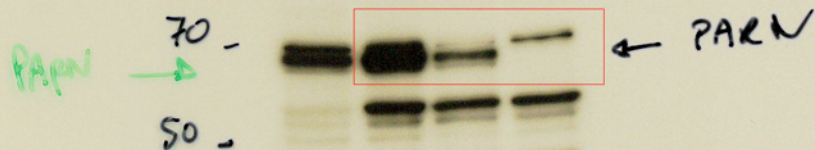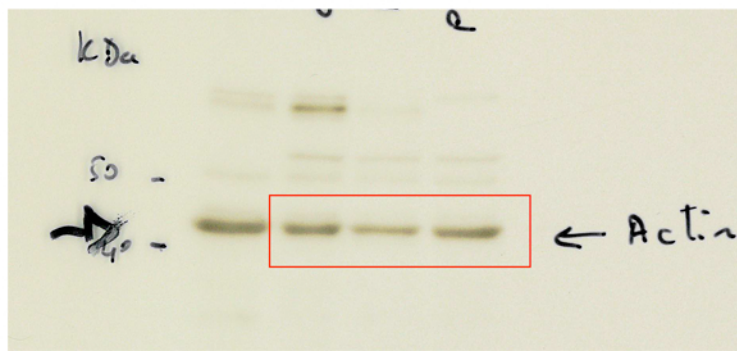

Supplement: Supplementary file 3 — Source Data for Figure 1 [file EMMM-11-e10201-s002.pdf]

Figure 3A

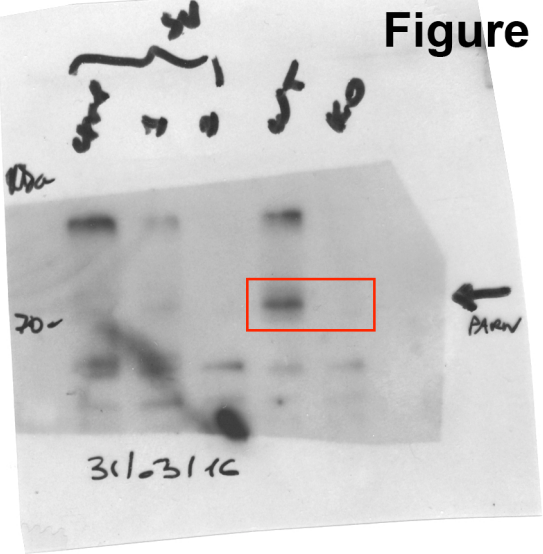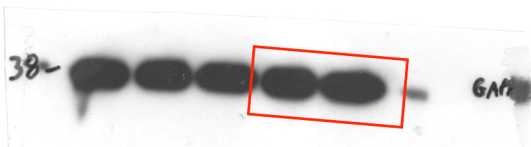

Supplement: Supplementary file 5 — Source Data for Figure 3 [file EMMM-11-e10201-s004.zip › emmm201810201-sup-0005-SDataFig3/emmm201810201-sup-0005-Fig3A.pdf]

# Figure 3F

Scan Li-Cor

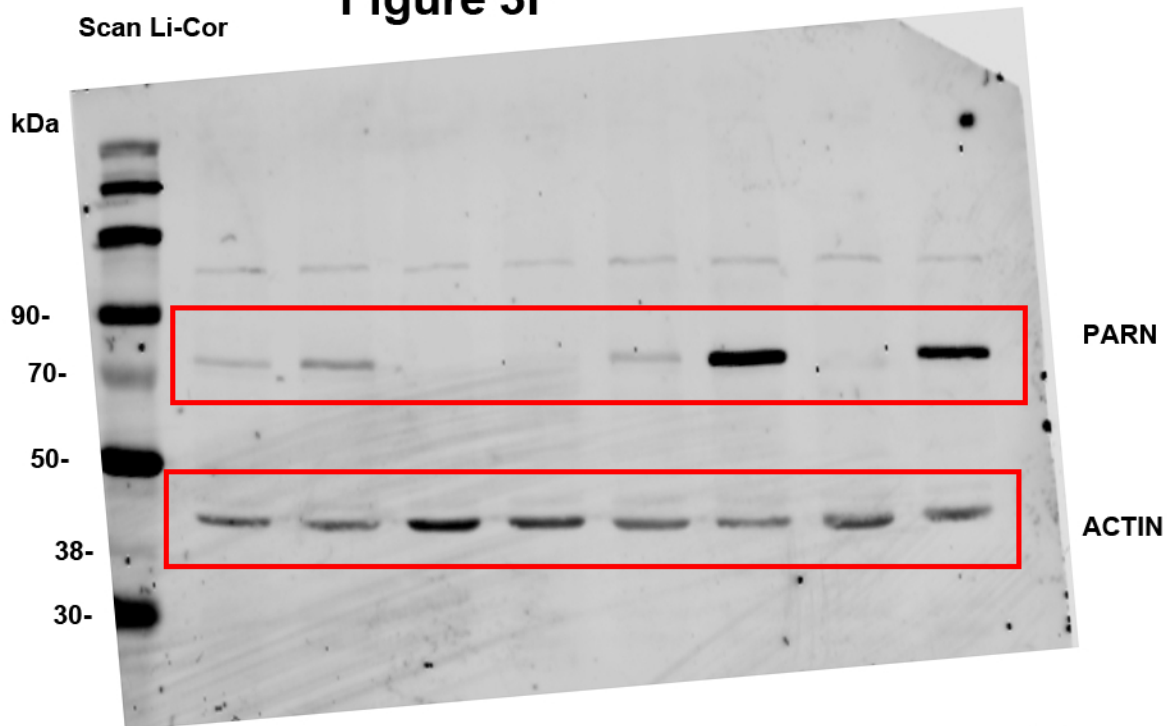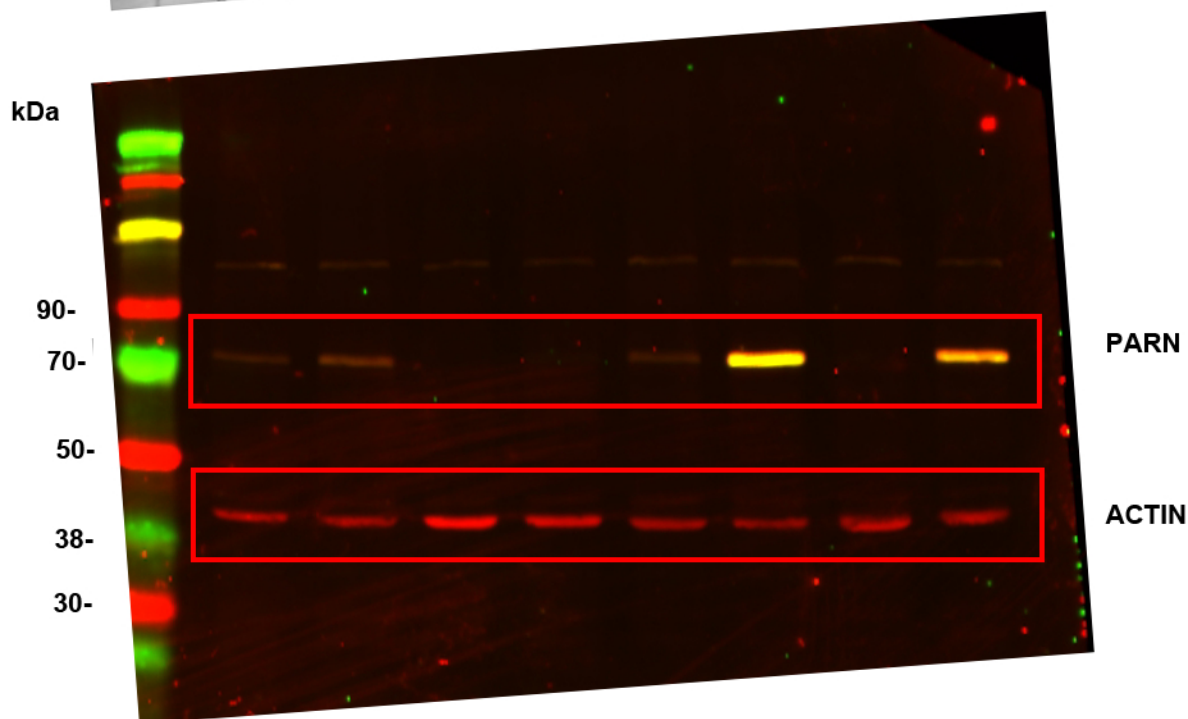

Supplement: Supplementary file 5 — Source Data for Figure 3 [file EMMM-11-e10201-s004.zip › emmm201810201-sup-0005-SDataFig3/emmm201810201-sup-0006-Fig3F.pdf]

Figure 3I

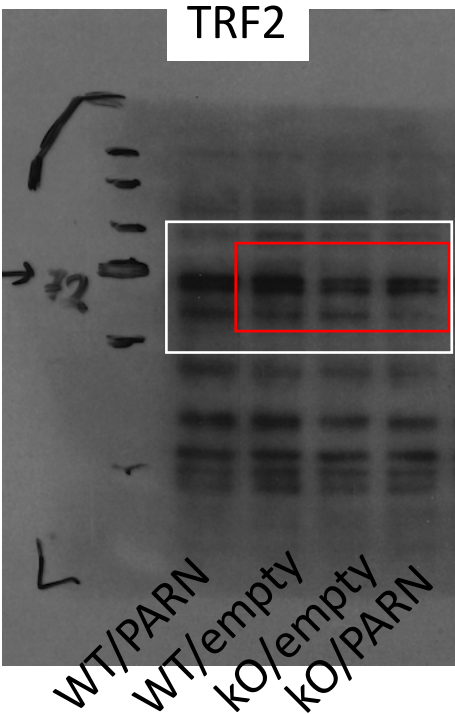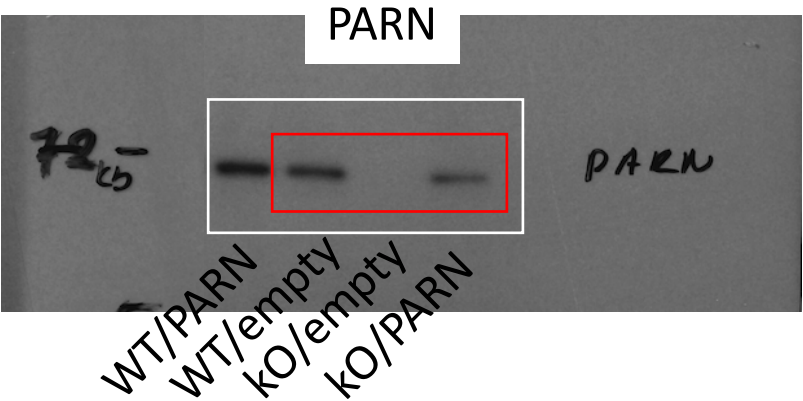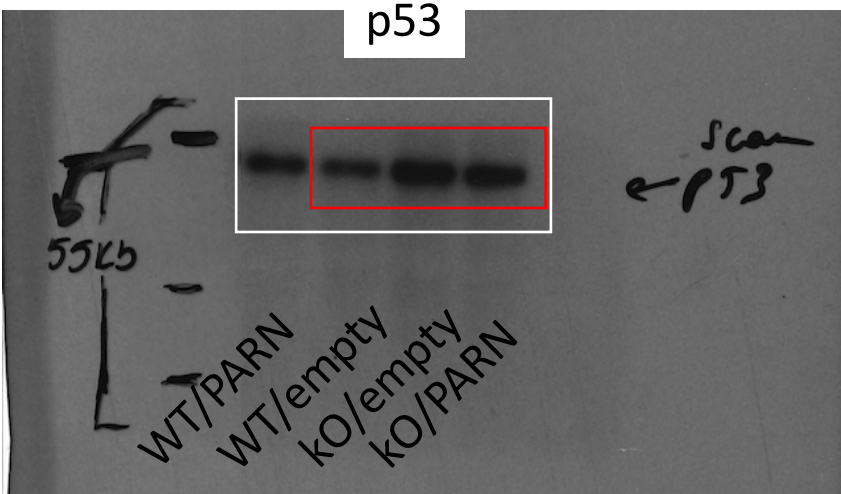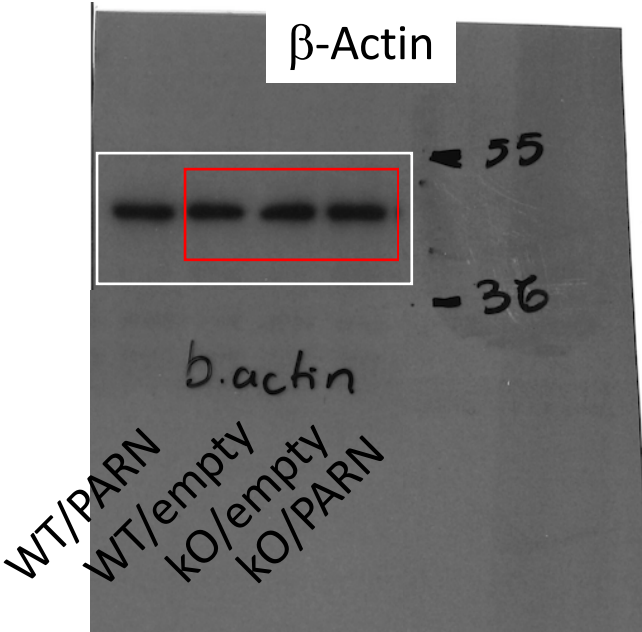

Supplement: Supplementary file 5 — Source Data for Figure 3 [file EMMM-11-e10201-s004.zip › emmm201810201-sup-0005-SDataFig3/emmm201810201-sup-0007-Fig3I.pdf]

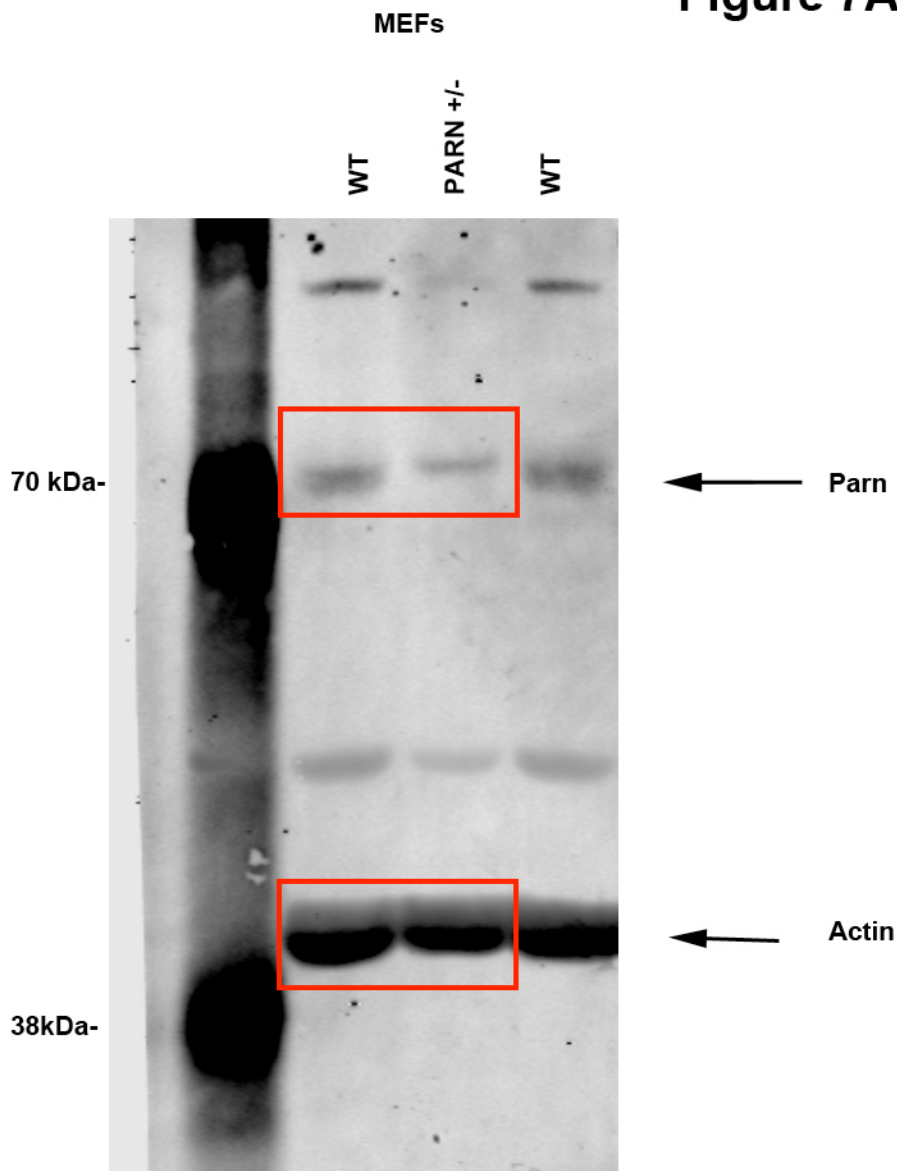

Supplement: Supplementary file 8 — Source Data for Figure 7 [file EMMM-11-e10201-s007.pdf]
